# A Case of Isolated Infragenicular Arterial Lesions Successfully Treated with Open Endarterectomy

**DOI:** 10.3400/avd.cr.20-00140

**Published:** 2021-03-25

**Authors:** Hiroko Okuda, Shinsuke Kikuchi, Atsuhiro Koya, Hidehiro Takei, Nobuyoshi Azuma

**Affiliations:** 1 Department of Vascular Surgery, Asahikawa Medical University, Asahikawa, Hokkaido, Japan; 2 Department of Pathology, Asahikawa Medical University Hospital, Asahikawa, Hokkaido, Japan

**Keywords:** endarterectomy, peripheral artery disease, localized infragenicular occlusive lesion

## Abstract

A 68-year-old man with a history of superior mesenteric arterial thromboembolism due to chronic atrial fibrillation had experienced intermittent claudication (IC) of his left leg for 3 years. Computed tomography angiography showed focal occlusive lesions in the left distal popliteal artery and proximal segments of the infrapopliteal arteries. Endarterectomy was performed for these localized arterial lesions, and a drastic symptomatic improvement of IC after revascularization was achieved. The endarterectomized segments remained patent for 4 years after the surgery. Endarterectomy could be a useful alternative to bypass surgery and endovascular therapy for the treatment of localized infragenicular arterial lesions.

## Introduction

Surgical endarterectomy is an established revascularization method for femoral atherosclerotic lesions.^1)^ Endovascular therapy (EVT) or bypass surgery is usually indicated for lesions of the infragenicular arterial segments; however, endarterectomy with a vein patch for atherosclerotic lesions was reported to provide acceptable clinical outcomes.^2–5)^ Herein, we report a case of localized infragenicular arterial lesions attributed to thrombosis and treated by endarterectomy without a vein patch. We will then discuss feasible indications for revascularization method.

## Case Report

**Figure 1:**
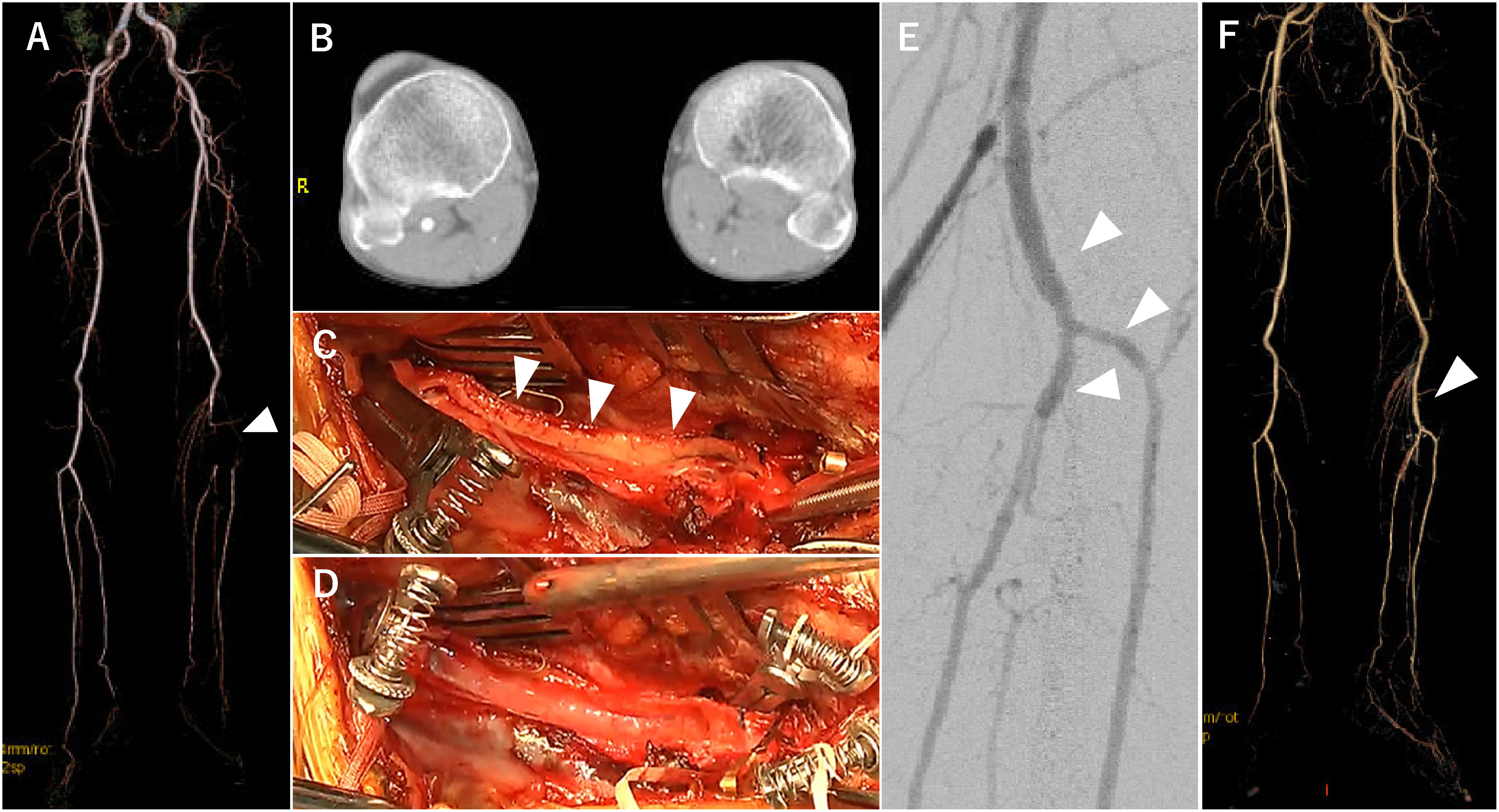
Fig. 1 Preoperative computed tomography angiography (CTA) showed occlusion of the left infragenicular arteries. The focal occlusive lesions involved the left distal popliteal artery and the trifurcation (arrowhead) (**A**). Axial imaging negated the possibility of popliteal artery entrapment and cystic adventitial disease of the popliteal artery (**B**). Intraoperative findings and postoperative CTA. Thickened walls were found in the infragenicular arteries, and soft and white thrombi were observed in the popliteal artery and tibioperoneal trunk (arrowheads) (**C**). Endarterectomy of 5 cm in length was carefully performed (**D**). The intraoperative angiography showed a well-recanalized popliteal artery, tibioperoneal trunk, and anterior tibial artery (arrowheads) (**E**). Postoperative CTA (1 week after the surgery) showed good revascularization of the infragenicular occlusive lesions (arrowhead) (**F**).

A 68-year-old man with a history of chronic atrial fibrillation (on warfarin therapy), hypertension, and diabetes mellitus was referred to our hospital for the treatment of activity-limiting intermittent claudication (IC) of the left leg that had continued for 3 years. After undergoing massive small bowel resection due to acute superior mesenteric arterial occlusion 3 years previously, he had experienced claudication symptoms. The ankle-branchial index of his left leg was 0.85, and enhanced computed tomography (CT) showed localized occlusive lesions in the distal popliteal artery and the trifurcation, indicating thrombus-induced limb ischemia, given that fewer atherosclerotic lesions were detected in other arterial segments (**Fig. 1A**). Furthermore, popliteal artery entrapment and cystic adventitial disease of the popliteal artery were excluded by axial imaging of enhanced CT (**Fig. 1B**). His activity-limiting IC did not improve despite additional antiplatelet and exercise therapies throughout the previous year. Preoperative ultrasonography disclosed no saphenous veins available for bypass grafting; therefore, we decided to perform revascularization with endarterectomy for the localized infragenicular arterial lesions.

The surgical procedure was performed under general anesthesia. The distal popliteal artery and the trifurcation were exposed by a medial approach. After a single longitudinal arteriotomy, the arterial walls were found to be thickened in these segments, and soft and white thrombi were observed in the popliteal artery and tibioperoneal trunk (**Fig. 1C**). Endarterectomy of 5 cm in length was carefully performed, and the intimal flaps of both proximal and distal edges were fixed with 8-0 polypropylene sutures to prevent dissection of the artery (**Fig. 1D**). The arteriotomy was sutured by direct closure with 7-0 polypropylene using a 3-mm blood vessel probe to ensure its adequate luminal patency. Intraoperative angiography showed a well-recanalized popliteal artery, tibioperoneal trunk, and anterior tibial artery (**Fig. 1E**). His postoperative course was uneventful, with good recanalization shown on CT angiography (**Fig. 1F**). Complete resolution of claudication was observed after the surgery, and the postoperative ankle-branchial index of his left leg recovered to 1.09. The site of endarterectomy has remained patent for 4 years postoperatively.

**Figure 2:**
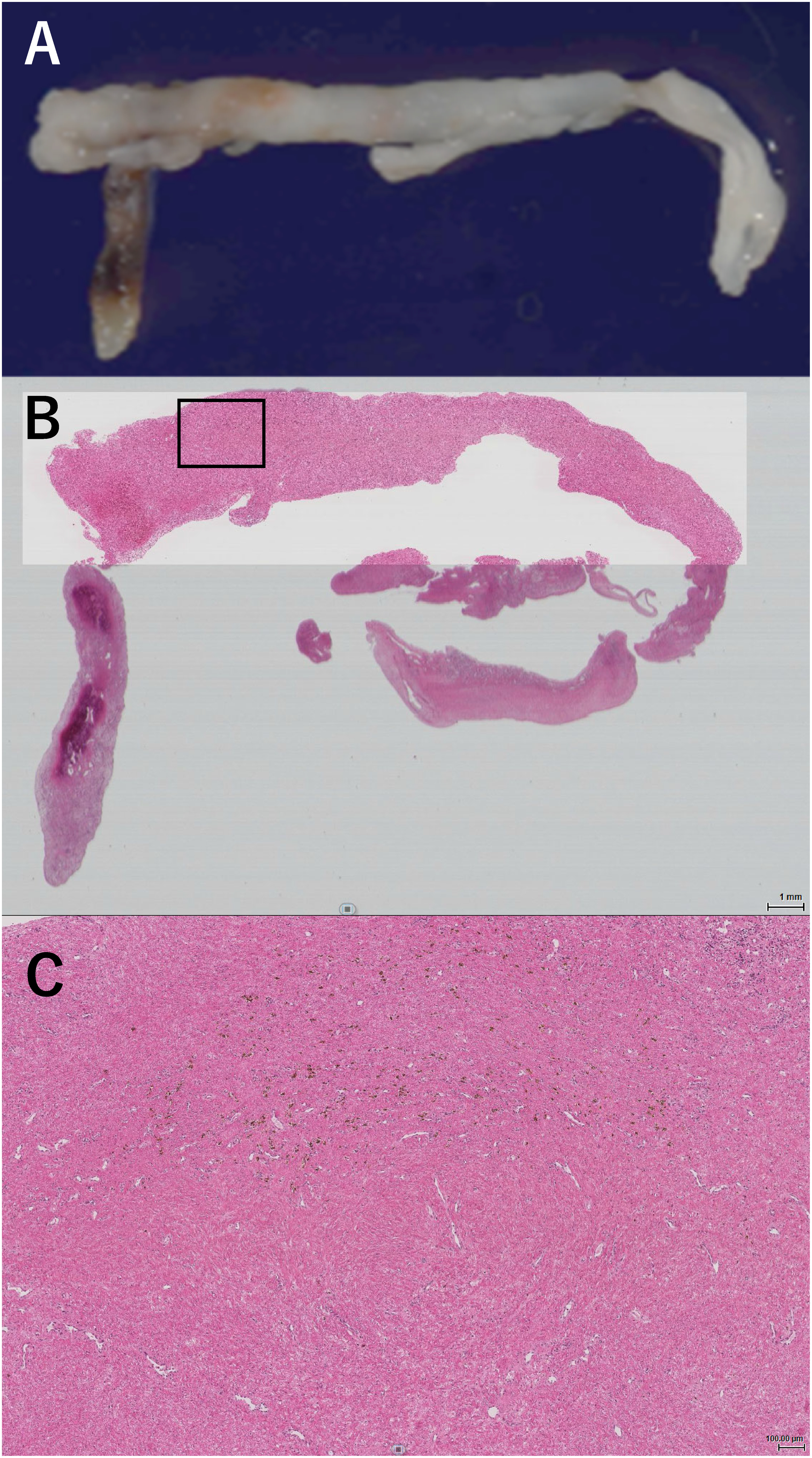
Fig. 2 The resected specimen consisted of white and focally red thrombi (**A**). Histology of the lesion demonstrated extensive fibrosis with hemosiderin deposition. No features associated with atherosclerosis were apparent (**B**, **C**).

The resected lesion was pathologically demonstrated significant fibrotic components with hemosiderin deposition. No features associated with atherosclerosis were found. These findings may be explained as pathologic changes of chronic thromboembolism (**Figs. 2A**–**2C**).

## Discussion

This case report shows technical success of endarterectomy for the infragenicular arterial lesions with long-term patency of the revascularization site. Little is known about the clinical outcomes of endarterectomy for infragenicular arterial lesions given the lack of studies on infragenicular endarterectomy in contrast to EVT or bypass procedures. However, some reports have suggested that infragenicular endarterectomy is safe and effective for localized atherosclerotic arterial disease. Nasr et al. reported short-term clinical outcomes of seven patients whose EVT-failed localized popliteal artery diseases were treated with endarterectomy through a posterior approach, with a 100% technical success rate and an 86% of 1-year patency rate.^2)^ Kumar et al. concluded that endarterectomy should be considered as a viable option for bypass procedures for short atherosclerotic popliteal and infragenicular arterial lesions. They basically used a patch for their endarterectomy including vein patching in 87% and prosthesis patching in 12%. Their primary patency rate was 89%, and their primary assisted patency rate after balloon dilation reached 96% at 3 years after surgery.^3)^ Abbas et al. emphasized the good results of open endarterectomy with a patch in short atherosclerotic occlusive artery of lower extremity including infragenicular segment.^5)^ Thus, several articles proved that endarterectomy is a feasible alternative procedure to bypass surgery and EVT in selected patients with localized disease developed in genicular segment. Regarding the current case, the bilateral lower extremity arteries other than the left infragenicular arteries were basically intact, with no atherosclerotic lesions, as shown in **Fig. 1**. The localized infragenicular occlusive lesions were most likely attributed to thromboembolisms based on his past history of chronic atrial fibrillation as well as angiographic findings. Therefore, endarterectomy was indicated as our first option for the present patient, who had no veins available for bypass grafting.

A point of concern is that residual lesions at one end of the endarterectomy site are associated with recurrent stenosis after surgery.^5)^ Therefore, arteriotomies of the endarterectomy site are mostly repaired with patch plasty to prevent restenosis, especially for patients in whom either inflow and outflow lesions exist or the arterial diameters are relatively narrow.^2–4)^ Vein patching is a common procedure, but the use of prosthetic patches should be carefully considered because infection of prosthetic patches following endarterectomy sometimes leads to severe complications, such as high mortality risk and major amputation.^6)^ The reason or indication for direct suture without a patch in the present case is that abnormalities in neither inflow nor outflow of the localized arterial lesions were present, which was probably due to embolic process. Adequate patency of the vascular lumen was also secured after direct suturing. In addition, there was probably little chance of recurrent stenosis after direct closure based on the histological findings of the resected thrombi, which were not associated with atherosclerosis.

These therapies are still associated with a low rate of long-term patency and further reinterventions despite the development of EVT and plaque debulking techniques such as laser-guided atherectomy for infragenicular occlusive lesions.^7,8)^ Therefore, endarterectomy of infragenicular localized lesions would be a more preferable option considering the high probability of restenosis after revascularization by EVT. It can also spare the long saphenous vein for use in future bypass surgery.

## Conclusion

We present a patient with localized infragenicular arterial disease that was treated with isolated endarterectomy. Endarterectomy of the infragenicular artery represents a viable option for selected patients with localized lesions using a judicious surgical technique, and this technique should be especially considered for patients with inadequate veins for bypass grafting.

## Additional Note

Patient consent for publication was obtained.
